# Chemical composition of axillary odorants reflects social and individual attributes in rhesus macaques

**DOI:** 10.1007/s00265-018-2479-5

**Published:** 2018-03-28

**Authors:** Brigitte M. Weiß, Marlen Kücklich, Ruth Thomsen, Stefanie Henkel, Susann Jänig, Lars Kulik, Claudia Birkemeyer, Anja Widdig

**Affiliations:** 1 Junior Research Group of Primate Kin Selection, Department of Primatology, Max Planck Institute for Evolutionary Anthropology, Deutscher Platz 6, 04103 Leipzig, Germany; 2 Research Group of Behavioural Ecology, Institute of Biology, Faculty of Life Sciences, University of Leipzig, Talstraße 33, 04103 Leipzig, Germany; 3 Department of Anthropology, University College London, Gower Street, London, WC1E 6BT UK; 4 Department of Developmental and Comparative Psychology, Max Planck Institute for Evolutionary Anthropology, Deutscher Platz 6, 04103 Leipzig, Germany; 5 Institute of Analytical Chemistry, Mass Spectrometry Research Group, University of Leipzig, Linnéstrasse 3, 04103 Leipzig, Germany; 6 German Center for Integrative Biodiversity Research (iDiv), Deutscher Platz 5E, 04103 Leipzig, Germany

**Keywords:** Body odors, GC–MS, *Macaca mulatta*, Old world monkey

## Abstract

**Abstract:**

Scents play an important role in the life of most terrestrial mammals and may transmit valuable information about conspecifics. Olfaction was long considered of low importance in Old World monkeys due to their relative reduction of olfactory structures and low incidence of scent-marking behavior but has been increasingly recognized for mediating social relationships in recent years. Yet, studies investigating the composition of their chemical cues remain scarce. In the present study, we analyzed the potential information content of chemicals present on the skin of rhesus macaques (*Macaca mulatta*). We collected axillary secretions from 60 animals of the semifree-ranging population on Cayo Santiago (Puerto Rico, USA) with precleaned cotton swabs from which the secretions were subsequently extracted and analyzed by gas chromatography–mass spectrometry. Rhesus macaque axillary odorants varied in their overall similarity and composition. This variation was attributable to differences in sex, group membership, and kinship and further appeared to reflect age and rank in parts of our sample. The compounds most strongly associated with this variation primarily comprised larger molecular weight aldehydes and steroids. Such compounds are considered to be perceivable by the primate olfactory system through close-range interactions or through breakdown into smaller molecules by bacterial fermentation. Overall, our results provide additional evidence that odors of Old World monkeys reflect a wealth of potential information about their carrier, which provides the basis for chemical communication via body odors; however, its use by conspecifics needs to be confirmed in bioassays.

**Significance statement:**

One prerequisite for olfactory communication is the presence of systematic variation in animal odors that is related to attributes such as age, sex, or kinship. The composition of odors has been examined in numerous mammals but, with the exception of humans, remains poorly understood in Old World monkeys and apes, taxonomic groups in which most species do not show scent-marking behavior. In the present study, we show that the composition of axillary secretions of an Old World monkey, the rhesus macaque, reflects sex, group membership, relatedness, and possibly also age and rank. This variation thus provides a basis for olfactory communication in Old World monkeys.

**Electronic supplementary material:**

The online version of this article (10.1007/s00265-018-2479-5) contains supplementary material, which is available to authorized users.

## Introduction

Scents are important mediators of social interactions between individuals (Wyatt 2014), with the relevance of olfactory communication for mammalian life being well established. For example, many mammalian species perform some types of scent-marking behavior using scent secretions (Rosell et al. 1998; Caspers and Voigt 2009), urine (Roberts et al. 2014; Vogt et al. 2014), and/or feces (Ghosal et al. 2012; Marneweck et al. 2017). The information transmitted by these scents has been investigated in numerous studies observing marking behavior and behavioral responses of individuals upon encountering natural or experimentally presented scents (Mares et al. 2011; Ghosal et al. 2012). Particularly in recent years, also the chemical composition of mammalian scents has become a focus of research on olfactory communication. In addition to numerous studies on model species in the laboratory (for instance in rodents, see Burger 2005), analyses applying analytical tools such as gas chromatography–mass spectrometry (GC–MS; see Charpentier et al. 2012) are applied to investigate odor cues in an increasing number of species, settings, and contexts. For instance, odors encode information about species, sex, and reproductive state in spotted and striped hyenas, *Crocuta crocuta* and *Hyaena hyaena* (Theis et al. 2013); age classes in Iberian wolves, *Canis lupus signatus* (Martín et al. 2010); or colony membership, relatedness, and heterozygosity in Antarctic fur seals, *Arctocephalus gazella* (Stoffel et al. 2015).

Among primates, a strong reliance on smell has been attributed to some taxonomic groups, but not to others. Strepsirrhine primates such as lemurs, for instance, have long been recognized to rely heavily on olfactory communication, as they show pronounced scent-marking behavior and are known to differentiate between scents depending on, e.g., individuals (Palagi and Dapporto 2006), familiarity, social status, and reproductive condition (Scordato and Drea 2007). Their scent secretions have been subject to a number of GC–MS studies that described variation in complexity and composition related to several social variables (delBarco-Trillo et al. 2012), genetic quality, and relatedness (Charpentier et al. 2008). Also, New World monkeys were shown to have well-developed olfactory capabilities (e.g., Laska and Hudson 1993). Similar to Strepsirrhines, their chemical cues indicate attributes such as reproductive state (Ziegler et al. 1993), familiarity (Smith et al. 1997), or individual identity (Smith 2006).

With the exception of humans, the role of olfactory communication has been relatively understudied in Old World monkeys (OWM) and apes. These have long been considered “microsmatic” (poor sense of smell) because of the relatively small size of their olfactory bulbs (Baron et al. 1983; but see Smith and Bhatnagar 2004) and reduced number of olfactory receptor genes (Gilad et al. 2004; but see Matsui et al. 2010). In addition, only few species of OWM or apes have been described to show scent-marking behavior. These include some of the guenons (Loireau and Gautier-Hion 1988; Freeman et al. 2012), the siamang (*Hylobates syndactylus*, Geissmann 1987), and the mandrill (*Mandrillus sphinx*, Feistner 1991), whose sternal scent gland secretions contain chemical cues of sex, age, and male rank (Setchell et al. 2010). Within the primate literature, human studies provide evidence that even in the absence of apparent scent-marking behavior, body odors may serve a communicative function in a large variety of contexts. In particular, axillary or other odors emanating from the skin may contain cues about enduring individual traits such as gender or identity, as well as about dynamic states such as health (reviewed in de Groot et al. 2017). These odors are strongly affected by microbes that are considered key contributors to body odors in humans and other mammals (Dormont et al. 2013; Theis et al. 2013). Notably, also a growing number of studies in OWM and nonhuman apes point at the relevance of olfactory communication irrespective of whether species show scent-marking behavior or not (reviewed in Drea 2015). For example, olfactory cues appear to play a role in mating behavior of chacma baboons, *Papio ursinus* (Clarke et al. 2009), and during feeding as well as social and sexual interactions in chimpanzees, *Pan troglodytes* (Matsumoto-Oda et al. 2007). Male stump-tailed macaques, *Macaca arctoides*, further appear to use olfactory cues to assess the reproductive status of females (Cerda-Molina et al. 2006). Hence, recent evidence points at a continued relevance and similar functionality of olfactory communication throughout the primate order despite the fact that vision has become the more important sense in OWM (Gilad et al. 2004). However, while behavioral evidence for the importance of olfactory communication in OWM and nonhuman apes is growing, the compounds mediating such chemical interactions (Wyatt 2014) remain poorly characterized. This current gap calls for more studies investigating the chemical composition of OWM scents and how these co-vary with social and life history attributes.

This study aimed at investigating the potential information content in the chemical composition of axillary odorants, i.e., the odors emanating from secretions and skin of the axillary region of rhesus macaques. Rhesus macaques live in multi-male, multi-female groups characterized by female philopatry (Gouzoules and Gouzoules 1987) and male dispersal (Lindburg 1969; Colvin 1983). They have a promiscuous mating system and breed seasonally. As in many other OWM, visual and acoustic communication play an important role in mediating rhesus social interactions (e.g., Higham et al. 2013; Pfefferle et al. 2015), while the role of olfactory communication is little explored. Yet, behavioral evidence indicates that rhesus macaques discriminate between social groups via olfactory cues alone (Henkel et al. 2015), and we can expect rhesus body odors to relate also to other relevant social or individual attributes, either as by-products of physiological processes and/or as signals evolved for communication. Therefore, we collected axillary secretions of semifree-ranging rhesus macaques from the island of Cayo Santiago (Puerto Rico) and combined individual chemical profiles obtained by GC–MS analysis with demographic and genetic data. In particular, we expected social and individual attributes such as sex, group membership, kinship, age, or dominance rank to affect the chemical composition of axillary odorants. We further identified candidate compounds that are most likely to relate to the respective attributes.

## Methods

### Study population

We conducted the study on semifree-ranging rhesus macaques living on Cayo Santiago, a 15.2-ha island off Puerto Rico, USA (details in Rawlins and Kessler 1986), managed by the Caribbean Primate Research Center (CPRC). During the study period (Jan to Mar 2011), the island was inhabited by approximately 1000 macaques all of which are direct descendants of 409 founder animals captured in 12 locations across Lucknow (India) in 1938 (Rawlins and Kessler 1986). However, pedigree analyses revealed no indications of inbreeding even after 75 years of genetic isolation (Widdig et al. 2017). Natural food found on the island such as foliage, fruits, insects, and soil, as well as monkey chow provisioned by the CPRC, contribute equally to the animals’ diet (Marriott et al. 1989).

### Study animals including information on sex, age, group, and kinship

For this study, we sampled 52 adult females and eight adult males. We considered only adult animals during the nonbreeding season to avoid any presumed hormonal influences of sex and life history stage on the chemical signals (Mitra 2003; Kean et al. 2011). Based on demographic records, females were 6–21 years (mean ± SD = 8.1 ± 3.3) and males 6–11 years (mean ± SD = 6.6 ± 1.8) old at the time of data collection. Females lived in five different social groups (group F 11, HH 12, KK 9, R 13, S 7), males in three of these groups (group F 2, KK 3, R 3). The birth group and the current group of residence were known for all animals from demographic data collected on a nearly daily basis by the CPRC since 1956. As female dominance rank in rhesus macaques is socially inherited and highly stable, we were able to determine female ranks (standardized from 0 to 1) from long-term observations updated with any changes resulting from births and deaths as described in Kulik et al. (2015). Male rank, on the other hand, is typically obtained by queuing whenever males enter a new group (Berard 1999), and we did not have sufficient behavioral data to determine male ranks at the time of data collection.

Kinship data were taken from a comprehensive genetic database available for this population, including 4641 animals that were genotyped for an average of 27.6 ± 1.6 microsatellite markers with both genetically confirmed maternity and assigned paternity for over 98% of animals genotyped (for details see Widdig et al. 2017). Specifically, our 60 study animals were genotyped on 28.0 ± 0.2 (mean ± SD) markers. Maternity determined from field observations was genetically confirmed for all study subjects, except for one for which we used the confirmed genetic mother. Using both a strict exclusion rule and a likelihood method confirming paternity at the 95% confidence level (see supplement of Widdig et al. 2017), paternity was assigned to all study subjects with the exception of one. In this remaining case, we assigned the best match as father (father had one mismatch, while other potential sires were excluded with two or more mismatches), which was confirmed at the 95% confidence level, too. The 60 animals used in this study had 54 different mothers, whereby 48 mothers contributed a single offspring and six mothers contributed two offspring to the data set. Similarly, the 60 focal animals had 45 different fathers, with 35 fathers contributing a single offspring, eight fathers two offspring, one father three, and another six offspring to the data set. Among the 60 focal animals, there were no parent–offspring or grandparent–grandchild dyads. Accordingly, our data set comprised the following kin categories: (i) maternal half-siblings (sharing the same mother but different fathers, *r* = 0.25, *N* = 6 dyads, all female), (ii) paternal half-siblings (sharing the same father but different mothers, *r* = 0.25, *N* = 26 dyads: 14 female–female, 10 male–female, 2 male–male), and (iii) distantly or unrelated individuals (*r* = 0.125, *N* = 37 dyads; *r* = 0.0625, *N* = 90 dyads; unrelated, *N* = 1611 dyads). We considered as unrelated dyads individuals sharing no ancestors up to and including the grandparental generation (*r* < 0.0625) based on available pedigree data for this population (Widdig et al. 2017).

### Sample collection

Altogether, this study included 132 samples collected from 60 rhesus macaques. Between January and March 2011, SH collected one to five samples per individual (mean ± SD = 2.2 ± 0.75) immediately after the animals were captured during the annual trapping season and anesthetized using an intramuscular injection of ketamine (10 mg/kg). Samples were collected with cotton swabs (precleaned with methanol and pentane) from the armpits, a body part that probably contains fewer contaminants (e.g., dirt or excreta) than odor samples from more exposed body parts. Swabs were repeatedly rubbed over the skin (Scordato and Drea 2007; Scordato et al. 2007; Lenochova et al. 2008) with ethanol-cleaned forceps for approximately 20 s and stored in precleaned glass vials (Rotilabo®). Samples were temporarily stored on the island at − 20 °C. Within a few hours, samples were transferred to the laboratory on the mainland of Puerto Rico in a transportable cooling bag and were subsequently stored at − 80 °C. As control, we collected and analyzed five blank cotton samples. All samples were given uninformative codes, which allowed us to conduct subsequent GC–MS analysis and processing of GC–MS data blind to the identity or other attributes of the sampled individuals.

### Gas chromatography–mass spectrometry analysis

Samples were extracted and analyzed in August and November 2012 (i.e., after 19–21 months of cold storage, which was previously shown to not affect chemical richness of or behavioral responses to odorants as reviewed in Drea et al. 2013). We used the solvent extraction method to extract chemical compounds from the swabs by adding 1.2 mL *n*-hexane to each cotton swab in its glass vial. After 10 min of incubation, repeatedly, 200 μL of the solvent was transferred to the microinsert (NeoLab®) of a 2-mL GC vial (Agilent®) and concentrated by evaporation to 60 μL (Stuart® Sample Concentrator). Then, 4 μL of each sample was injected into the GC (HP 6890 Series GC System) coupled to an MS (HP 5973 MSD) operated in electron-impact ionization mode at 70 eV. A DB35-MS column (30 m long, 0.25 mm id, and 0.25 μm film, J&W Fisher®) was used with helium as the carrier gas. The GC oven program started from 35 °C (held for 2 min) to 320 °C with an increase of 10 °C/min. Solvent delay was set to 7 min, the ion source temperature to 250 °C, and the scan range between *m/z* 50 and 550. The samples were analyzed within the same batch to ensure the comparability of the obtained signal intensities. The instruments’ performance was monitored by a QC standard on a daily basis.

### Identification and classification of substances

To determine which of the chemical components indeed derived from the macaques (and thus could be considered as endogenous) or were likely to be contaminants or other exogenous compounds, we used library identification (NIST 08, National Institute of Standards and Technology, USA) followed by manual confirmation of all peaks per chromatogram after automatic peak detection with AMDIS (Stein 1999); for details, see Birkemeyer et al. (2016). We treated compounds as contaminants or exogenous if they appeared in the control blank samples in similar or higher concentrations than in the rhesus samples, which included compounds deriving from plants due to the biological origin of the cotton swabs (cf. Birkemeyer et al. 2016). We further excluded compounds if they are known to be GC column bleed appearing repeatedly with similar and regular MS fragmentations (Drea et al. 2013) as well as signals that were tentatively identified as a known system contamination (such as softeners and stabilizers). Compounds not present in blanks but known to derive from plants (such as phytosterols) or other parts of the environment were excluded from further analysis, as these probably originated from dirt or plant particles present on the skin of the macaques during sampling. Finally, compounds quantifiable in fewer than three samples were not considered further as we assumed that they had no general relevance. As a result, we used 21 out of 140 detected compounds for further analysis and discarded the remaining compounds.

### Statistical analysis

For the analysis of chemical profiles obtained from axillary secretions, we used two different approaches. First, we compared the overall similarity between chemical profiles, (Stoffel et al. 2015; Weiß et al. 2018) and second, we investigated the difference in compound composition between chemical profiles in relation to our test predictors (Weiß et al. 2018). Unless stated otherwise, we conducted all analyses for the 60 individuals of both sexes. Due to the bias in sample size towards females, we also repeated the analyses for the subset of 52 females only.

#### Similarity of chemical profiles

For all analyses conducted in this study, we used R version 3.2.4 (R Core Team 2016). We assessed effects of sex and group membership at the time of sample collection on the overall similarities between chemical profiles using nonparametric analysis of similarity (ANOSIM). This test compares the similarity of samples within one test category with the similarity of samples in different test categories. Profile similarities were computed as pairwise Bray–Curtis indices, which are a widely adopted similarity measure that computes a form of standardized absolute deviation between samples based on presence/absence as well as abundance of each compound while not making distributional assumptions (Clarke 1999). Bray–Curtis indices were calculated from standardized (peak area/sum of all 21 peak areas × 100), log(*x* + 1)-transformed peak areas. We ran additional ANOSIMs to compare the similarities of chemical profiles of maternal or paternal half-siblings with the profiles of distantly or unrelated individuals (dyads not sharing the same parent, *r* < 0.25). ANOSIMs were implemented using a customized R script (written by LK) that was adapted from the ANOSIM implemented in the R package “vegan” (Oksanen et al. 2016). The customized script returned *p* values corrected for repeated measures by permuting the samples within the individual or condition to which they belong. To relate age and rank similarities between pairs of individuals to their chemical profile similarities, we computed Mantel tests in the R package “vegan” (Oksanen et al. 2016) based on individual means of chemical profiles. The analysis for dominance rank was only computed for females, as we did not have systematic observations of dominance rank for males.

#### Comparison of chemical profile composition

For a detailed comparison of chemical profile composition between sexes, groups, and ages, we performed a linear mixed model (LMM) using the R package “lme4” version 1.1–11 (Bates et al. 2015). We used an approach that vectorizes the multi-variate data matrix (Jamil et al. 2013) and therefore fitted standardized peak areas of each sample (*n* = 132) and compound (*n* = 21) as Gaussian response (using an arcsine and log(*x* + 0.01) transformation to improve model fit). Sex, social group, and age (in years) were fitted as fixed effects, with age being z-transformed to facilitate model convergence and interpretation (Schielzeth 2010). In the female subset, we further included dominance rank (determined as described above) as an additional fixed effects predictor. To avoid problems of pseudo-replication and heteroscedastic variance introduced by vectorizing the data matrix, we included the matrix rows and columns (the samples and compounds) as random factors (Jamil et al. 2013). We further fitted the identities of the sampled individual, its mother and father as random effects. As we do not expect any fixed effect to affect all compounds in the same manner but some more and others less, we fitted the random slopes of all fixed effects predictors within “compound” as our actual test predictors. This allowed us not only to assess the general importance of a given predictor for axillary odorant composition, but also to identify the compounds most characteristic for that predictor (showing the steepest slopes, Weiß et al. 2018).

The model fulfilled the assumption of normally distributed residuals and showed no indication of collinearity (all variance inflation factors < 1.5, determined with R package “car,” see Fox and Weisberg 2011). An inspection of residuals against fitted values indicated a slight boundary effect (arising from the standardized areas being bounded between 0 and 100); however, model estimates were stable when levels of the random effects were omitted. Following Forstmeier and Schielzeth (2011), we first determined the significance of the full model by comparing it to a null model excluding the random slopes using a likelihood ratio test [LRT, (Barr et al. 2013), R function “anova” with test argument set to “Chisq” (Dobson 2002)]. In a similar manner, we then assessed the significance of the individual random slopes with LRTs by comparing the full model against models lacking the respective slopes of interest. In addition, we extracted the slope estimates per compound and predictor from the model results. We derived the compounds most affected by a given predictor as those with the steepest slopes, with a particular focus on the compounds whose absolute slope estimate was at least one standard deviation above the average absolute slope estimate for the respective predictor (Weiß et al. 2018).

##### Data availability

The data set generated and analyzed during the current study is included as electronic supplementary material.

## Results

The 132 analyzed samples from 60 rhesus macaques contained 5–21 (mean ± SD 13.60 ± 3.97) of the 21 compounds considered for statistical analysis.

### Similarity of chemical profiles

**Fig. 1:**
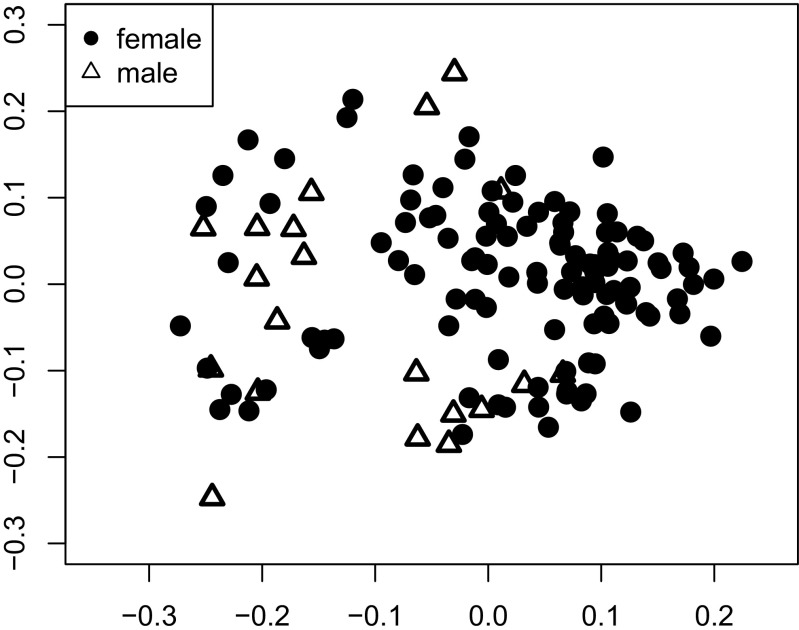
Two-dimensional nonmetric multi-dimensional scaling plot of chemical profiles of female (black circles) and male (white triangles) rhesus macaques based on Bray–Curtis indices. The axes are dimensionless; symbols in close proximity indicate similar chemical profiles

**Fig. 2:**
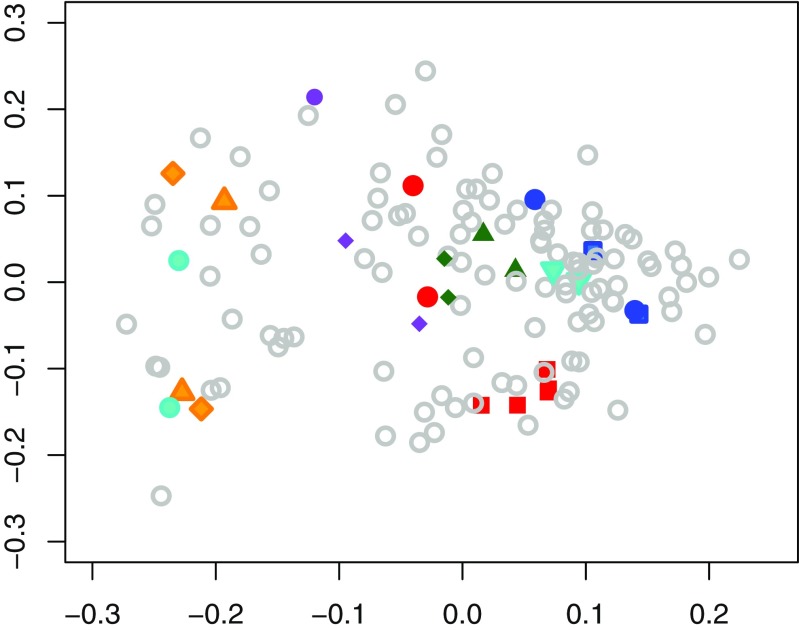
Two-dimensional nonmetric multi-dimensional scaling plot of chemical profiles of male and female rhesus macaques based on Bray–Curtis indices. Colored symbols depict samples from individuals sharing the same mother, with different individuals being encoded by different symbols within a given color. Open gray circles depict samples from individuals without maternal half-siblings in the data set. The axes are dimensionless; symbols in close proximity indicate similar chemical profiles

In the full data set (i.e., including males and females), the overall similarity between chemical profiles was greater for individuals of the same sex than for the different sexes (ANOSIM *r* = 0.342, *p* = 0.001; Fig. 1). It was also greater for animals living in the same than in different social groups, although this difference was less pronounced (ANOSIM *r* = 0.105, *p* = 0.025). Moreover, maternal half-siblings had more similar odor profiles than more distantly or unrelated individuals (ANOSIM *r* = 0.52, *p* = 0.001; Fig. 2). We detected no such pattern for paternal half-siblings in relation to distantly or unrelated individuals (ANOSIM *r* = 0.21, *p* = 0.634). We further detected no relationship between the overall similarity of odor profiles and age differences between individuals (Mantel test, *r* = 0.015, *p* = 0.426).

Similar to the full data set, when testing only females, the overall similarity between odor profiles was greater for individuals living in the same than in different social groups (ANOSIM *r* = 0.149, *p* = 0.006), and maternal half-sisters had more similar odor profiles than more distantly or unrelated females (ANOSIM *r* = 0.482, *p* = 0.002). Unlike the full data set, however, paternal half-sisters tended to have more similar profiles than distantly or unrelated females (ANOSIM *r* = 0.362, *p* = 0.06). We detected no relationship between the overall similarity of odor profiles and age or rank differences between females (Mantel test, age *r* = − 0.053, *p* = 0.777; rank *r* = − 0.059, *p* = 0.929).

### Comparison of chemical profile composition

**Fig. 3:**
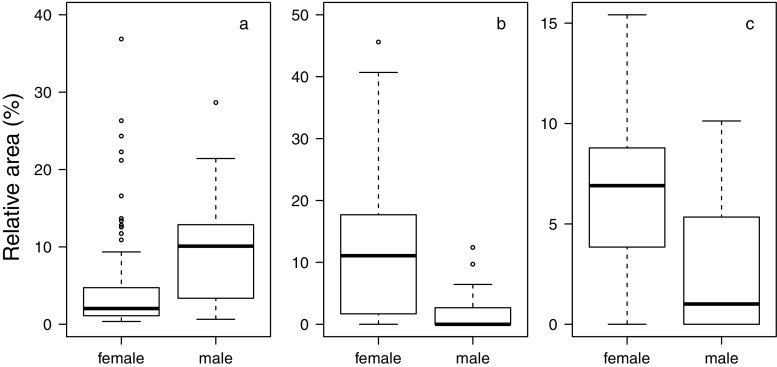
Relative areas of compounds most characteristic for sex differences in rhesus macaque axillary odorants. **a** RT 29.47, which was tentatively identified as cholesta-3,5-diene. **b** An unknown compound at RT 31.70. **c** RT 32.26, which was tentatively identified as 5α-cholest-7-en-3β-ol. Boxplots show medians and first and third quartiles. Lower (upper) whiskers are located at the larger (smaller) value of the minimum (maximum) × value or the first (third) quartile minus (plus) 1.5 × interquartile range

**Fig. 4:**
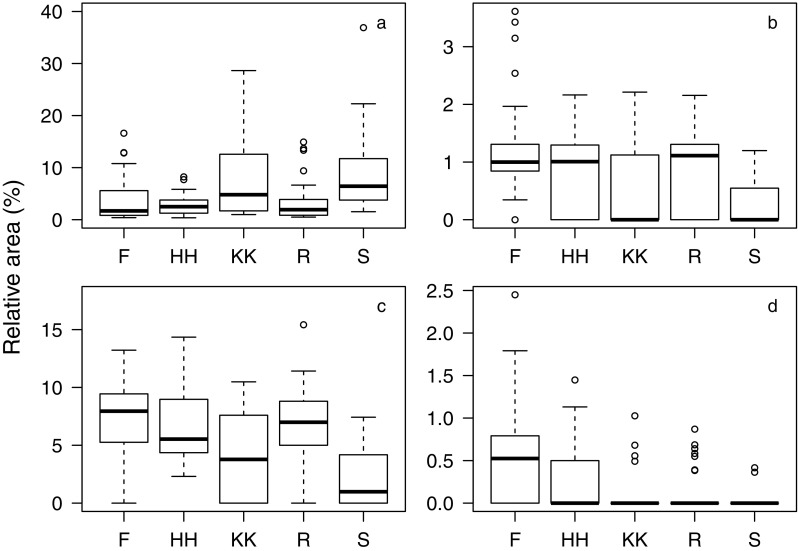
Relative areas of compounds most characteristic for group differences in male and female rhesus macaque axillary odorants. **a** RT 29.47, which was identified as an unspecified steroid. **b** RT 31.87, which was identified as a cholesteryl- or cholestenylester. **c** RT 32.26, which was tentatively identified as 5α-cholest-7-en-3β-ol. **d** RT 32.51, tentatively identified as 14-heptadecenal. Boxplots show medians and first and third quartiles. Lower (upper) whiskers are located at the larger (smaller) value of the minimum (maximum) × value or the first (third) quartile minus (plus) 1.5 × interquartile range

**Fig. 5:**
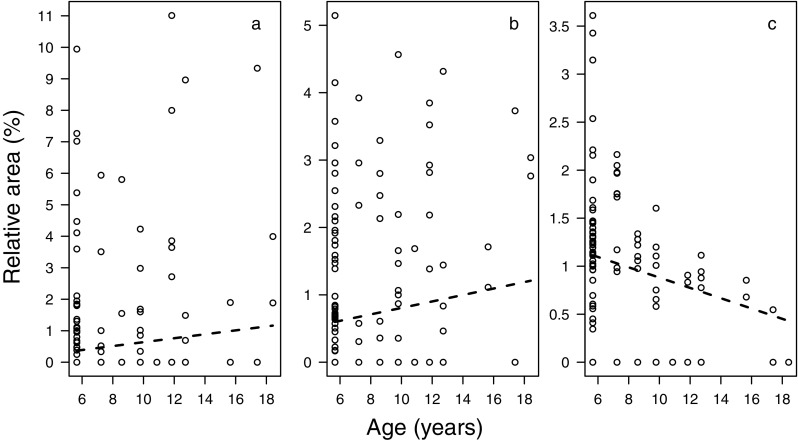
Relative areas of compounds most characteristic for age differences in male and female rhesus macaque axillary odorants. Dashed lines show the random slope estimates for the respective compounds, derived from a model with sex and group centered to a mean of zero (at their average). **a** RT 18.20, which was tentatively identified as nonadienal. **b** RT 21.40, tentatively identified as octadecanal. **c** RT 31.87, which was identified as a cholesteryl- or cholestenylester

A detailed analysis of sample composition in males and females showed that the animals’ sex, group membership, and age affected the relative abundance of compounds in a nonrandom fashion (Table 1). Specifically, sex differences were most pronounced for three compounds from which two could be tentatively identified as steroids, while the third was unknown (mass spectrum provided in Fig. S1 of Online Resource 1). The unknown and one steroid were more abundant in females and the other steroid more abundant in males (Table 2; Fig. 3). Both steroids involved in sex differences further showed pronounced variation between animals from different social groups along with another steroid compound and an aldehyde (Table 2; Fig. 4). Age differences in the relative abundance of compounds were best described by an increase of two tentative aldehydes and a decrease of a steroid with older age (Table 2; Fig. 5).

**Table 1:** Results of likelihood ratio tests assessing the effects of all (full vs. null model) and individual random slopes on sample composition

	Full data set			Female data subset		
	*χ* ^2^	*df*	*p*	*χ* ^2^	*df*	*p*
Full-null model	146.67	25	< 0.001	130.85	17	< 0.001
Sex	41.96	3	< 0.001			
Social group	69.22	21	< 0.001	70.55	15	< 0.001
Age	12.63	1	0.0004	0.42	1	0.518
Rank				36.21	1	< 0.001

**Table 2:** Retention time (RT), tentative identification, chemical structure, and random slope estimates for each compound retained for statistical analysis. Slope estimates per predictor are derived from the random slopes model of the full data set with standardized peak area as response variable. Positive slope estimates for sex indicate higher values in females, while negative values indicate higher values in males. Slope estimates for group are given as the largest difference observed between groups. Positive slopes for age indicate an increase and negative slopes a decrease of the respective compound with older age. Slope estimates marked in italics mark the compounds with the strongest effects (> 1 SD above average slope estimates) for the respective predictor

RT	Tentative ID	Chemical Class	Sex	Group	Age
16.59	Methyl hexadecane	Alkane	− 0.2068	0.1442	− 0.012
18.10	Farnesane	Sesquiterpene	− 0.1675	0.3507	0.092
18.20	Nonadienal	Aldehyde	− 0.1908	0.2047	*0.207*
21.40	Octadecanal	Aldehyde	− 0.1037	0.3451	*0.160*
22.30	9,12-Octadecadienoic acid, methyl ester	Carboxylic acid ester	− 0.0963	0.1423	− 0.012
22.34	Eicosanol	Alcohol	− 0.0629	0.1967	0.034
25.40	unknown		0.3534	0.4108	− 0.127
27.97	Squalene	Terpene	0.191	0.1517	0.007
29.38	(3β)-Cholesta-4,6-dien-3-ol	Steroid	− 0.4515	0.4917	− 0.037
29.47	Cholesta-3,5-diene or cholesteryl/cholestenylester	Steroid or steroid ester	*− 0.5952*	*0.7419*	0.008
29.86	1-Octacosanol	Alcohol	0.149	0.6042	− 0.055
31.54	Cholesterol	Steroid	− 0.1272	0.1683	− 0.064
31.65	(5β)-Cholestan-3-one	Steroid	0.171	0.2983	− 0.087
31.70	Unknown		*0.9151*	0.4538	0.115
31.87	Cholesteryl- or cholestenylester	Steroid	0.0018	*0.8313*	*− 0.168*
32.26	“(3β,5α)-Cholest-7-en-3-ol	Steroid	*0.735*	*1.0231*	− 0.096
32.51	14-Heptadecenal	Aldehyde	0.3201	*0.8861*	− 0.019
32.87	Cholesta-3,5-dien-7-one	Steroid	− 0.5317	0.6051	− 0.045
33.25	Cholest-4-en-3-one	Steroid	0.1531	0.2857	− 0.005
35.02	Cholesteryl- or cholestenylester	Steroid ester	0.0394	0.2221	0.066
35.21	Cholesteryl- or cholestenylester	Steroid ester	− 0.4952	0.4531	0.035

As in the full data set, group membership of females affected the relative abundance of compounds in a nonrandom fashion, while we no longer detected an age effect on chemical composition when considering only females (Table 1). In addition, female rank affected the relative abundance of certain compounds (Table 1). The four compounds that showed the largest variation between the groups in the full data set were also among the five compounds that showed the largest variation in the data subset on the 52 females (Online Resource Table S1). The fifth compound contributing most to group differences among the 52 females could not be identified. Rank differences were best described by a decrease in an alcohol and an increase by a steroid ester and an unknown compound with increasing rank (Online Resource Table S1).

## Discussion

The chemical composition of rhesus macaque axillary odorants showed variation in its overall similarity and specific composition that was attributable to differences in sex, group membership, kinship, and potentially also to age and rank. The compounds most strongly associated with this variation predominantly comprised large molecular weight aldehydes, esters, and steroids.

### Sex differences

The present study suggests that sex differences are present in axillary odorants of rhesus macaques. Despite the imbalanced sex ratio in our data set, the detected sex differences are in line with previous results of GC–MS studies that described a sex-specific composition of scent secretions, urine, or feces of various mammals, including primates (Setchell et al. 2010; Kean et al. 2011; Marneweck et al. 2017). The compounds differing the most between male and female rhesus macaques comprised two steroids. Although this result should be confirmed in future GC–MS studies, steroids are reasonable candidates to act as potential cues for sex differences, as they affect and are affected by (sex-specific) behavior throughout the animal kingdom (Neave 2007). Furthermore, steroids are well known to occur in mammalian sebaceous and apocrine glands and appear to affect the chemistry of secretions as well as bacterial communities (Theis et al. 2013). Although the mere presence of sex-specific cues does not necessarily mean that animals use this potential information, behavioral studies in several species provide evidence that animals perceive and attend to sex-specific odors (Cross et al. 2014; Gilfillan et al. 2017).

### Group differences

A group specificity of mammalian scents primarily has been described in behavioral studies conducting bioassays (Mares et al. 2011; Gilfillan et al. 2017), while only few studies addressed the chemical properties underlying perceived group differences. For instance, odors appear to encode information about group membership in mandrills (Vaglio et al. 2016), Bechstein’s bats (*Myotis bechsteinii*; Safi and Kerth 2003), and meerkats (*Suricata suricatta*; Leclaire et al. 2017). Furthermore, in owl monkeys (*Aotus nancymaae*), a New World monkey with pronounced scent-marking behavior, chemical profiles of body odors distinctly differed between family groups (MacDonald et al. 2008). To what extent this distinction resembled relatedness or actual group differences remained unclear in owl monkeys, however, the bacterial communities contributing to social odors in meerkats varied between groups independent of relatedness (Leclaire et al. 2014). In rhesus macaques, group membership is less confounded with kinship than in owl monkeys because groups also contain a large number of unrelated individuals (Pfefferle et al. 2014) and our analysis indeed detected some group-specificity in rhesus chemical profiles. Although differences in the overall similarity of axillary odorants were not very pronounced, differences in chemical composition persisted even when close relatedness (*r* ≥ 0.25) was controlled. It should also be noted that these group differences are perceived by unrelated conspecifics, as previously shown in a bioassay study by Henkel et al. (2015) in the same study population. The compounds distinguishing the different social groups best comprised three steroids, suggesting that hormonal differences arising from group-specific behavior and rank differences may be at the base of the detected differences in axillary secretions. Group differences were further associated with variation in an aldehyde, which is one of the main volatile compound classes in scent secretions of hyenas, where bacterial fermentation appears to mediate group-specific odors (Theis et al. 2013).

### Kinship

In line with results in ring-tailed lemurs (*Lemur catta*; Boulet et al. 2009), similarities between chemical profiles co-varied with kinship in the present study. In this study, the sampled rhesus macaques comprised six dyads of maternal half-sisters, whose chemical profiles were significantly more similar to each other than to more distantly or unrelated individuals. Furthermore, paternal half-sisters tended to have more similar chemical profiles than distantly or unrelated individuals, although this effect was less pronounced than for maternal half-sisters and was nonsignificant when using the full data set containing 14 half-sisters, two half-brothers, and 10 half-sibling dyads of mixed sex. Yet, this hints at a certain similarity also between paternal half-siblings, but one that may be inconsistent due to masking effects such as sex differences or subtle environmental differences that arise from residing in different social groups (as was the case in our analysis). Given the behavioral preferences for paternal siblings shown in rhesus macaques (Widdig et al. 2001, 2016), the potential of olfactory cues for kin recognition should be further evaluated for both sexes in future studies.

In fact, similarities in whole chemical profiles of maternal kin may be partly explicable by a shared environment, as maternal kin typically live in the same social group. However, maternal kin similarities were considerably more pronounced than group similarities, suggesting that they are not entirely due to a shared group membership. Rather, similarities between maternal kin may be more pronounced than among paternal kin due to the closer bonds among maternal kin (for the study population: Widdig et al. 2001), where frequent grooming and other direct contact facilitate a cross-contamination with odor-producing bacteria or other odor sources (Theis et al. 2012; Leclaire et al. 2014). In sum, these results provide some indication for a chemical kin label that may be the product of genetic similarities in combination with a shared social environment. Similar to the family group label in owl monkeys (MacDonald et al. 2008), rhesus axillary odorants may thus contain a cue to both kin and group membership.

### Age differences

While we detected no effect of the age difference between pairs of individuals on their profile similarity, the chemical composition explored in our second analytical approach varied significantly in relation with absolute age when considering both males and females in the analysis. These age effects were best described by an increase of two aldehydes tentatively identified as nonadienal and octadecanal and as such showed a similar pattern as described in humans: a characteristic “old people smell” was suggested to relate to an age-dependent functionality of skin glands (Mitro et al. 2012) and, among others, an increased production of two aldehydes, nonenal (Haze et al. 2001) and nonanal (Gallagher et al. 2008) with older age. Despite this similarity in patterns, our result needs to be treated with caution, as the described age effect disappeared when considering only females in the analysis. Hence, age effects in females may have been so weak or masked that they were only detectable with the additional power of the male samples; alternatively, they may represent a statistical artifact.

### Rank differences

A differentiation of dominant and subordinate individuals based on animal odors has been described in various mammals (reviewed in Scordato et al. 2007). Social rank was also reflected in the composition of glandular secretions in male mandrills (Setchell et al. 2010; Vaglio et al. 2016), but not in ring-tailed lemurs (Scordato et al. 2007). A bioassay study in our study population had previously described a tendency for the monkeys placing their nose close to a scent source for longer if the odor donor was from a higher-ranking group than the inspecting animal, while no difference in the time spent sniffing was found with respect to group ranks (Henkel et al. 2015). In the present study, we assessed individual rather than group ranks and while overall similarities between axillary samples did not reflect rank differences between females, the relative abundance of compounds varied with female ranks. As rhesus offspring rank directly below their mothers while residing in the same group (Datta 1988), female rank and relatedness are confounded in this female-philopatric species. We controlled for close kinship in our analysis but cannot exclude that the observed rank effect was partly due to shared maternal relatedness between females of similar rank. Furthermore, attributes such as body condition or health frequently correlate with rank (Sapolsky 2005; Giles et al. 2015), but these were not systematically collected in the present study. Accordingly, a promising avenue for future research will be to try to disentangle effects truly reflecting individual ranks from those of correlated attributes.

### Relevance for chemical communication

As expected, given the used sampling methodology, the compounds identified in rhesus odor samples predominantly were larger molecular weight compounds (Birkemeyer et al. 2016). These were classically assumed to be perceived by the vomeronasal organ (VNO) when animals lick an odor source, while the main olfactory epithelium (MOE) is specialized in detecting the smaller, more volatile compounds (Dulac and Torello 2003). A functional VNO is thought to be lacking in catarrhine primates (Maier 1997), but it is becoming increasingly clear that the functional distinction between MOE and VNO is less discrete than long assumed (see overview in Charpentier et al. 2013; Wyatt 2014). Hence, larger molecules may also be partly detected by the MOE (Wyatt 2014), e.g., when entering the nose through the suction created by sniffing at close range and may actually provide longer-lasting information than low molecular weight compounds (Alberts 1992). Furthermore, bacteria contribute to body odors by metabolizing the larger, semi-, and nonvolatile compounds into smaller, more volatile ones (Archie and Theis 2011).

Hence, even though the chemical senses of OWM and apes may be more tuned for the perception of volatile compounds with low molecular weight, the larger molecular weight compounds identified as carriers of information may still be perceived. The perceptibility per se is supported by the bioassay study by Henkel et al. (2015), which used the same sampling methodology as in the present study and showed that rhesus macaques were able to use the chemical information present in samples taken with cotton swabs for differentiating between social groups. Whether conspecifics can also use the systematic differences in axillary odorants to deduce an individual’s sex, age, and kinship and whether these differences indeed relate to the compounds identified as most likely candidates in this study will need to be assessed in bioassay studies. Furthermore, we used a very strict preselection regime of compounds to avoid assigning contaminants or environmental components as potential olfactory cues and might have identified more compounds covering a wider chemical range in a less conservative approach. Future studies using different methodological approaches to assess the information content of more volatile compounds (Kücklich et al. 2017; Weiß et al. 2018) would complement the results of the present study and contribute to a better understanding of chemical communication in OWM and primates in general.

In conclusion, the chemical composition of rhesus axillary odorants relates to a range of social and individual attributes, namely sex, group membership, kinship, and possibly also age and dominance rank. This variation is a prerequisite for olfactory communication and may thus point at a greater importance of olfaction for rhesus macaques, and OWM in general, than previously appreciated. To what extent rhesus macaques indeed use this information and how it is perceived should be subject to future investigations.

## Electronic supplementary material

Online Resource 1 Additional Fig. of mass spectrum and Table for the data subset containing only females (*n* =112 samples of 52 individuals) (PDF 178 kb)
