# Supplementary material for: Chemical composition of axillary odorants reflects social and individual attributes in rhesus macaques
Source: Behav Ecol Sociobiol. 2018 Mar 28;72(4):65. doi: 10.1007/s00265-018-2479-5 (PMC5871651; doi:10.1007/s00265-018-2479-5)
Supplement: Supplementary file 1 — Additional Fig. of mass spectrum and Table for the data subset containing only females (n =112 samples of 52 individuals) (PDF 178 kb) [file 265_2018_2479_MOESM1_ESM.pdf]

Supplement to " **Chemical composition of axillary odorants reflects social and individual attributes in rhesus macaques "** in Behavioral Ecology and Sociobiology,

Weiß BM*, Kücklich M, Thomsen R, Henkel S, Jänig S, Kulik L, Birkemeyer C, Widdig A

* correspondence: Research Group of Behavioural Ecology, Institute of Biology, Faculty of Life Sciences, University of Leipzig, Talstraße 33, 04103 Leipzig, Germany, email: brigitte.schloegl@uni-leipzig.de

Table S1 Retention time (RT), tentative identification, chemical structure and random slope estimates for group and rank differences in the data subset on 52 female rhesus macaques. Slope estimates are given as the largest difference observed between groups. Compounds marked in bold showed the steepest slope estimates for group differences in the full data set. Positive slopes for rank indicate an increase and negative slopes a decrease of the respective compound with higher rank, with slope estimates marked in bold indicating the steepest slopes. Estimates for age are not presented because it did not significantly affect sample composition.

**RT**	**tentative ID**	**structure**	**group**	**rank**
16.59	Methyl hexadecane	Alkane	0.1005	-0.0751
18.10	Farnesane	sesquiterpene	0.6294	-0.0623
18.20	Nonadienal	Aldehyde	0.4058	0.1078
21.40	Octadecanal	Aldehyde	0.5730	-0.0920
22.30	9,12-Octadecadienoic acid, methyl ester	carboxylic acid ester	0.1627	-0.1532
22.34	Eicosanol	alcohol	0.1675	**-0.1918**
25.40	unknown		0.8192	0.0516
27.97	Squalene	terpene	0.0578	0.1630
29.38	Cholesta-4,6-dien-3-ol, (3β)-	steroid	0.4552	-0.1268
**29.47**	**Cholesta-3,5-diene *or* Cholesteryl/Cholestenylester**	**steroid *or* steroid ester**	**0.7611**	-0.1689
29.86	1-Octacosanol	alcohol	0.5787	0.0489
31.54	Cholesterol	steroid	0.1866	-0.0675
31.65	(5β)-Cholestan-3-one	steroid	0.4555	-0.0115
31.70	unknown		0.4083	**0.2871**
**31.87**	**Cholesteryl- *or* Cholestenylester**	**steroid**	**0.9291**	0.0866
**32.26**	**Cholest-7-en-3-ol, (3β,5α)-**	**steroid**	**1.3892**	0.1472
**32.51**	**14-Heptadecenal**	**aldehyde**	**0.7414**	0.0593
32.87	Cholesta-3,5-dien-7-one	steroid	0.5710	-0.1072
33.25	Cholest-4-en-3-one	steroid	0.2247	0.0977
35.02	Cholesteryl- *or* Cholestenylester	steroid ester	0.1905	**0.1745**
35.21	Cholesteryl- *or* Cholestenylester	steroid ester	0.5661	-0.1674

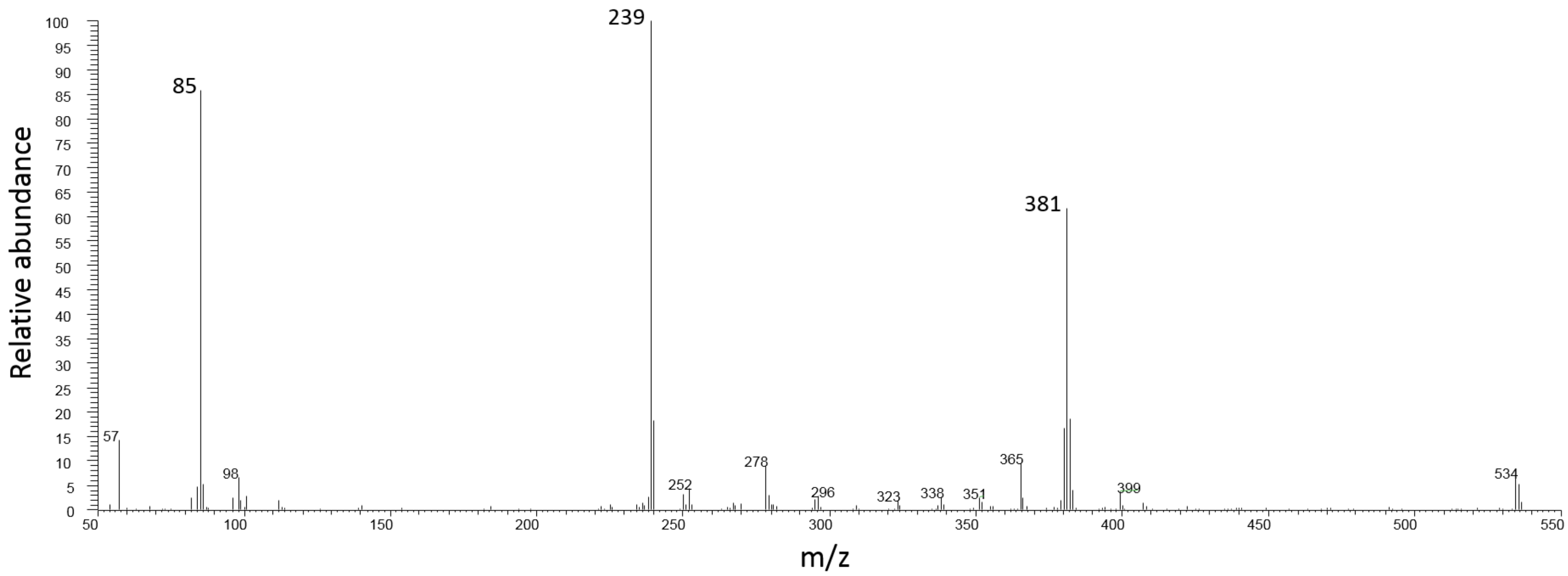

Figure S1 Two-sided background subtracted mass spectrum of unknown compound at retention time 31.7 min.
